# Botulism

**Published:** 1919

**Authors:** 


**Botulism.** By Ernest C. Dickson. *Bulletin of the Canadian Medical Corps*, September, 1918.

Botulism or Allantiasis, the old "Wurstfergiftung" of Southern Germany, has been recognized as a serious type of food poisoning since the early part of the 19th century. Until 1904, however, it was thought to be exclusively a type of meat poisoning, and the fact that botulinus infection may be caused by canned vegetables or fruit was not proved until 1914. As a result of the experiments made at that time, it has been established beyond doubt that the toxin of Bacillus Botulinus may be formed in media prepared from peas, beans, corn, asparagus, artichokes, peaches, pears, apricots and prunes; moreover, it has been ascertained that, in most cases, the vegetables or fruit were home-canned.

The poisoning is produced by a bacterial toxin formed in the food before it is eaten. This toxin is ingested with the food and absorbed unchanged into the blood stream; it is a peculiar characteristic type of thrombus formation in the blood vessels of practically all organs.

The first symptoms are: fatigue, disturbances of vision, vertigo and incoordination of muscular movements, usually appearing 4 to 8 hours after ingestion. Difficulty of speech, strangling spells and digestive troubles, with general weakness and occasional muscular paralysis, follow. The temperature is subnormal. There is also marked hyperemia of most organs, and usually hemorrhages occur in the meninges, lungs and serous surfaces. The intoxication, in case of recovery, subsides after 4 to 8 days and is followed by a long, tedious convalescence. The mortality has been 40% for Europe, and 65 to 70% for America.

The treatment should, in short, be as follows: purgation, washing of the stomach and lower bowel, and stimulation if required. Food and laxatives should preferably be administered by means of stomach tubes. Antitoxic serum is of little value, unless given very early.

The author again mentions the fact that botulinus infection was, in the majority of cases, caused by home-canned foodstuffs, and outlines the following principles:

(1) Only the best methods of home canning should be recommended.

(2) The housewife should be instructed in the methods of preventing poisoning by canned products.

(3) Home-canned food showing signs of spoilage should on no account be tasted.

(4) All home-canned food should be boiled before it is eaten.

